# Lipidomic alteration of plasma in cured COVID-19 patients using ultra high-performance liquid chromatography with high-resolution mass spectrometry

**DOI:** 10.1042/BSR20204305

**Published:** 2021-03-05

**Authors:** Yunpeng Bai, Wendong Huang, Yaocai Li, Changchun Lai, Sumei Huang, Guangwen Wang, Yuemei He, Linhui Hu, Chunbo Chen

**Affiliations:** 1 Center of Scientific Research, Maoming People’s Hospital, Maoming 525000, China; 2 Department of Critical Care Medicine, Maoming People’s Hospital, Maoming 525000, China; 3 Department of Cardiology, Maoming People’s Hospital, Maoming 525000, China; 4 Department of Infectious Diseases, Maoming People’s Hospital, Maoming 525000, China; 5 Department of Clinical Laboratory, Maoming People’s Hospital, Maoming 525000, China; 6 Biological Resource Center of Maoming People’s Hospital, Maoming 525000, China

**Keywords:** COVID-19, differential analysis, high-resolution mass spectrometry, lipidomics, SARS-CoV-2

## Abstract

**Background:** The pandemic of novel coronavirus disease 2019 (COVID-19) has become a serious public health crisis worldwide. The symptoms of COVID-19 vary from mild to severe among different age groups, but the physiological changes related to COVID-19 are barely understood.

**Methods:** In the present study, a high-resolution mass spectrometry (HRMS)-based lipidomic strategy was used to characterize the endogenous plasma lipids for cured COVID-19 patients with different ages and symptoms. These patients were further divided into two groups: those with severe symptoms or who were elderly and relatively young patients with mild symptoms. In addition, automated lipidomic identification and alignment was conducted by LipidSearch software. Multivariate and univariate analyses were used for differential comparison.

**Results:** Nearly 500 lipid compounds were identified in each cured COVID-19 group through LipidSearch software. At the level of lipid subclasses, patients with severe symptoms or elderly patients displayed dramatic changes in plasma lipidomic alterations, such as increased triglycerides and decreased cholesteryl esters (ChE). Some of these differential lipids might also have essential biological functions. Furthermore, the differential analysis of plasma lipids among groups was performed to provide potential prognostic indicators, and the change in signaling pathways.

**Conclusions:** Dyslipidemia was observed in cured COVID-19 patients due to the viral infection and medical treatment, and the discharged patients should continue to undergo consolidation therapy. This work provides valuable knowledge about plasma lipid markers and potential therapeutic targets of COVID-19 and essential resources for further research on the pathogenesis of COVID-19.

## Introduction

Novel coronavirus disease 2019 (COVID-19) is a new type of coronavirus first reported in Wuhan in late 2019 [1]. COVID-19 is caused by severe acute respiratory syndrome coronavirus 2 (SARS-CoV-2) [2]. Coronaviruses are a large family of viruses that are known to cause colds, severe acute respiratory syndrome [3], and Middle East respiratory syndrome (MERS) [4,5]. The early symptoms of COVID-19 infection are similar to flu virus infections and mainly manifest as fatigue and muscle soreness. Some patients may have gastrointestinal symptoms, such as abdominal pain and diarrhea [6]. Patients with worsening symptoms tend to have dyspnea, chest tightness, shortness of breath, respiratory distress, and even pulmonary interstitial changes. In severe cases, sepsis, septic shock, coagulation dysfunction, and renal failure may occur [7]. SARS-CoV-2 mainly attacks the lower respiratory tract. Some patients may develop life-endangering acute respiratory distress syndrome (ARDS). The invasion of SARS-CoV-2 in the liver, muscles, gastrointestinal tract, lymph nodes, and the heart has also been found or proposed [8,9].

Moreover, the mortality rate of COVID-19 in critically ill patients can reach more than 60%, especially in the elderly, which puts tremendous pressure on achieving successful treatment outcomes [10]. Therefore, we need to understand better the host metabolic response associated with the disease, expand our knowledge about its pathogenesis, and provide evidence for potential therapeutic strategies. However, the physiological changes related to COVID-19 are not yet fully understood, and effective treatment of severe patients remains speculative because of limited understanding of SARS-CoV-2 pathogenesis.

Lipids have various critical biological functions in cellular barriers, signal transduction, material transport, energy storage, cell differentiation, and apoptosis [11,12]. Lipids can be divided into classes and subclasses according to their head group and the type of connection between the head group and acyl chains [13]. The diversities in the head group and acyl chain of lipids lead to many individual lipid molecular species [14], including fatty acyls (FAs), glycerolipids (GLs), glycerophospholipids (GPs), sphingolipids (SPs), sterol lipids (STs), saccharolipids (SLs), prenol lipids (PRs), and polyketides (PKs). Furthermore, lipids have a highly complex composition and structure and have a wide range of concentrations. Since the pathway and network of lipid metabolism have been widely studied, changes in lipid content can reveal changes in enzyme activity and gene expression patterns simultaneously. In addition, abnormal lipid metabolism can lead to many human diseases, including diabetes, obesity, cancer, and neurodegenerative diseases [15].

As a powerful analytical tool, lipidomics can systematically describe lipid alterations of complex biological samples in response to internal or external subtle perturbations [16]. It has become an exciting field with broad prospects and has been widely used in drug research and development, molecular physiology and pathology, functional genomics, nutrition, and health issues [17]. Previous studies have demonstrated significant changes in the lipidome in human plasma caused by various diseases, like Ebola virus disease (EVD) [18,19]. Compared with healthy volunteers, the lipid metabolism of EVD patients had changed dramatically. With the recovery process, the difference in lipid molecules gradually decreased, which implied that the patients recovered. SARS-CoV-2 can induce characteristic molecular changes detected in patients’ sera using proteomic and metabolomic technologies [20,21]. These changes may provide valuable knowledge about plasma biomarkers and insight for the treatment of COVID-19 patients. Here we performed the untargeted lipidomics analysis of plasma samples collected from a cohort of cured COVID-19 patients and healthy controls. We found abnormal lipid metabolism in cured COVID-19 patients when they were about to discharge the hospital, indicating that viral infection and drug treatment affected the patients’ systemic metabolism. Although the sample size was relatively small and the age factor was not fully considered during the comparison process, this work could provide valuable knowledge of COVID-19-related plasma biomarkers and potential therapeutic targets. It is an essential resource for further studies of COVID-19 pathogenesis, suggesting that these discharged patients were not fully recovered from the physiological effects of COVID-19. The results of the present study will contribute to the understanding of COVID-19 and its clinical treatment strategies.

## Materials and methods

### Materials and reagents

HPLC-grade acetonitrile (ACN), methanol (MeOH), and isopropanol (IPA) were purchased from Thermo Fisher Scientific, U.S.A. HPLC-grade methyl-tert-butyl ether (MTBE), ammonium formate, and formic acid were purchased from Sigma–Aldrich (St. Louis, MO, U.S.A.). Ultrapure water was obtained by a Milli-Q system (Millipore, Billerica, MA). SPLASH internal standards (330707, SPLASH™ Lipidomix Mass Spec Standards) were purchased from Avanti Polar Lipids (Alabaster, U.S.A.), including lyso-phosphatidylcholine (LPC) 18:1 (d7), 25 μg/ml; lyso-phosphatidylethanolamine (LPE) 18:1 (d7), 5 μg/ml; phosphatidylcholine (PC) 15:0-18:1 (d7), 160 μg/ml; phosphatidylethanolamine (PE) 15:0-18:1 (d7), 5 μg/ml; phosphatidylglycerol (PG) 15:0-18:1 (d7), 30 μg/ml; phosphatidylserine (PS) 15:0-18:1(d7), 5 μg/ml; phosphatidylinositol (PI) 15:0-18:1 (d7), 10 μg/ml; phosphatidic acid (PA) 15:0-18:1 (d7), 7 μg/ml; sphingomyelin (SM) d18:1-18:1 (d9), 30 μg/ml; cholesterol (d7), 100 μg/ml; ceramide (Cer) 18:1 (d7), 350 μg/ml; monoglyceride (MG) 18:1 (d7), 2 μg/ml; diglyceride (DG) 15:0-18:1 (d7), 10 μg/ml; and triglyceride (TG) 15:0-18:1 (d7)-15:0, 55 μg/ml

### Patients and sample preparation

The research has been carried out in accordance with the World Medical Association Declaration of Helsinki and approved by the Maoming People’s Hospital Ethics Committee. The written informed consents were provided by all subjects from January 2020 to March 2020. Diagnosis of SARS-CoV-2 infection was based on the New Coronavirus Pneumonia Prevention and Control Program (6th edition) issued by the National Health Commission of China, and SARS-CoV-2-positive patients were enrolled in the study after diagnosis. Blood samples (≤3 ml) were collected when the patient’s disease was diagnosed as negative and met the discharge criteria. The basic information of the cured patients is displayed in Table 1, including age, gender, the days from onset to hospitalization and symptoms. The cured patients were divided into two groups: one group was the rehabilitation of severe symptoms or elderly patients (the SE group). The other group was composed of young people with mild symptoms (the MY group) who were considered to have no underlying diseases. It can be seen from Table 1 that the age of SE groups was older than 40 years and the members of MY groups were younger than 40 years. Healthcare staff younger than 35 years was recruited as the healthy control group from Maoming People’s Hospital, who were thought to have no underlying diseases as well. None of them had previously been infected with SARS-CoV-2, and their throat swabs and serological testing were negative for SARS-CoV-2. Because of the large age span of SE group, it was not easy to find suitable healthy controls to compare with SE group only, so the same healthy cohort was chosen to compare with SE and MY groups. All blood samples were collected by potassium-EDTA blood collection tubes according to the biocontainment procedures of SARS-CoV-2-positive specimen treatment, and the plasma was obtained through 3000 rpm/min centrifugation for 10 min.

**Table 1:** Basic information of 14 cured COVID-19 patients

No.	Age	Sex	Onset to hospitalization (days)	Symptom	Classification
1	68	Female	29	Mild	SE
2	60	Male	27	Mild	SE
3	40	Male	39	Severe	SE
4	53	Male	17	Severe	SE
5	53	Male	24	Severe	SE
6	41	Male	15	Severe	SE
7	33	Female	10	Mild	MY
8	18	Female	11	Mild	MY
9	26	Male	12	Mild	MY
10	22	Male	8	Mild	MY
11	38	Male	12	Mild	MY
12	34	Male	15	Mild	MY
13	16	Male	11	Mild	MY
14	23	Male	23	Mild	MY

Lipid extraction was conducted according to the MTBE method [22]. Briefly, 200-μl plasma samples were added to 1.2 ml of chilled mixture of methanol/MTBE/water (4:5:5, v/v/v). Samples were incubated on ice for 1 h and vortexed for 1 min every 15 min. After centrifugation (3000 rpm/min, 5 min), 200 μl supernatant of each sample was transferred to new tubes and dried under nitrogen flow at room temperature. Samples were re-suspended in 300 μl IPA (−20°C precooling) with 10 μl SPLASH internal standards and then centrifuged at 12000 rpm/min for 20 min at 4°C. The supernatant was transferred to the sampling bottles for UPLC-high-resolution mass spectrometry (HRMS) analysis, and 10 μl of each sample was mixed into the quality control (QC) samples to evaluate the repeatability and stability of the LC-MS process [23].

### UPLC-HRMS measurement of lipidomic compounds

Five microliter of samples was injected and separated using a CSH C18 column (1.7 μm, 2.1 × 100 mm, Waters, U.S.A.) on a Waters 2D UPLC system. Flow rate was 0.35 ml/min while column temperature was 55°C. The mobile phases for positive ion mode consisted of (A) 60% ACN in water with 10 mM ammonium formate and 0.1% formic acid and (B) 10% ACN in IPA with 10 mM ammonium formate and 0.1% formic acid. The mobile phases for negative ion mode consisted of (A) 60% ACN in water with 10 mM ammonium formate and (B) 10% ACN in IPA with 10 mM ammonium formate. Linear gradient was as follows: 0–2 min, 40–43% B; 2–2.1 min, 43–50% B; 2.1–7 min, 50–54% B; 7–7.1 min, 54–70% B; 7.1–13 min, 70–99% B; 13–13.1 min, 99–40% B; 13.1–15 min, 40% B.

Mass spectrometry was performed with a Thermo Scientific Q Exactive™ benchtop Orbitrap mass spectrometer equipped with heated ESI source in positive and negative modes (Thermo Scientific, San Jose, CA). Data acquisition consisted of a 200–2000 *m/z* full Fourier transform mass spectrometry (FTMS) scan event with the resolution at 70000, AGC target of 3e6, and maximum ion injection time (max IT) of 100 ms. The samples were also analyzed with data-dependent tandem MS acquisition methods in order to obtain lipidomic information at the mass range of 100–2000 *m/z* with a resolution at 17500, AGC target of 1e5, and max IT of 50 ms. For data-dependent acquisitions (DDAs), the method was set to analyze the three most intense ions, and normalized collision energy (NCE) were set at 15, 30, 45 eV. The spray voltage was set to 3.80 kV for positive ion mode and 3.20 kV for negative ion mode. High-purity nitrogen (99.9%) was used as the sheath gas with a flow rate of 40 arbitrary units and auxiliary gas flow rate of 10 arbitrary units. The capillary temperature was set at 320°C and auxiliary gas heater temperature 350°C [24]. In order to provide more reliable experimental results and reduce the systematic error, the samples were randomly sorted and one QC sample was inserted into every ten samples for data QC.

### Lipid identification and statistical analysis

The acquired base peak chromatograms (BPCs) and mass spectra obtained from UPLC-ESI-HRMS were exported as raw files by Xcalibur (Thermo Scientific, San Jose, CA). LipidSearch software v.4.1 (Thermo Fisher Scientific, U.S.A.) was used to extract the three-dimensional dataset, including *m/z*-values, retention times, and peak areas for lipid identification and peak alignment. The following parameters were used for lipid identification and peak extraction: the product was selected as the identification type, the quality deviation of the precursor ion and product ion in the library was 5 ppm, and the response threshold was set as the relative response deviation of product ion (5.0%). The quantitative parameter was set to calculate the peak areas of all identified lipids, and the mass deviation of peak extraction was set to 5 ppm. The filter was set as the top rank, all isomer peak, FA priority, M-score set as 5.0, c-score set as 2.0, and the identification level was selected as ‘A,’ ‘B,’ ‘C,’ or ‘D.’ All lipid categories were selected for identification; the additional forms of positive ion mode for [M+H]^+^, [M+NH_4_]^+^, [M+Na]^+^, and the additional forms of negative ion mode for [M−H]^−^, [M−HCOO]^−^, were selected, respectively. All lipids identified were peak-aligned, and results not marked as ‘reject’ were considered for further analysis. The peak alignment method was set as the mean. The retention time deviation was set as 0.1 min. The peak filtering was set as New Filter, top rank, all isomer peak, and the identification level was selected as ‘A,’ ‘B,’ ‘C,’ or ‘D.’

The original data exported by LipidSearch were imported into metaX [25] for data preprocessing and subsequent analysis. The data preprocessing content includes: (1) deleted lipid molecules missing more than 50% of QC samples and more than 80% of experimental samples; (2) K-nearest neighbor [26] algorithm (KNN algorithm) was used to fill the missing value; (3) The Probabilistic Quotient Normalization (PQN [27]) was used to normalize data and obtain relative peak area. (4) Lipid molecules whose coefficient of variation (CV) of relative peak area was greater than 30% in all QC samples were deleted. Log2 transformation of data conversion and Pareto scaling with mean centering for normalization were calculated with multivariate statistical analysis for the pair of comparison groups, including unsupervised principal component analysis (PCA) for the overall distribution and supervised partial squared discriminant analysis (PLS-DA) for the potential differential lipids. Two hundred response permutation testing (RPT) was performed to avoid model over-fitting, and the variable importance of projection (VIP) of each lipid molecule was obtained. The univariate analyses were also performed with Fold-Change analysis (FC) and *t* test (Student’s *t* test). FC-value, *P*-value and t.test_p.value_BHcorrect were separately obtained by FC analysis, *t* test and False Discovery Rate (FDR) correction.

The Metaboanalyst online tool (https://www.metaboanalyst.ca/) was applied for the pathway analysis identified highly enriched metabolic pathway of differential lipids via the Kyoto Encyclopedia of Genes and Genomes (KEGG) database. A *P*-value <0.05 identified significantly changing pathways and was used for subsequent analysis.

## Results

### Patients and analytical performance of the MS instrument

High lipidomic coverage was achieved by untargeted lipidomics of the plasma samples from cured COVID-19 patients and healthy controls using Q Exactive HRMS coupled with a UPLC system. Blood samples were collected at Maoming People’s Hospital from cured COVID-19 patients, confirmed by the laboratory nucleic acid test of SARS-CoV-2. Until 30 September 2020, a total of 14 patients with COVID-19 were treated in Maoming People’s Hospital, who were further divided into the SE group (six cured cases with severe symptoms or elderly patients) and the MY group (eight cured cases of relatively young people with mild symptoms). For comparison, blood samples from five healthy young volunteers were collected as the control group for two batches of mass spectrometry experiments. However, the limitation of the present study could be the direct comparison of SE group (over 40 years old) with the healthy control with age less than 35, since the change of plasma lipids might be related to age.

The QC samples from two MS batches were separately inserted into each analytical batch to evaluate the reproducibility and stability of the analytical system. As shown in Supplementary Figure S1, QC samples from each group were closely gathered in the center of the PCA (Supplementary Figure S1A for the SE group, Supplementary Figure S1B for the MY group), indicating that the instrument was in satisfactory condition and the MS signal was stable during the whole sample detection process of the two MS batches. Furthermore, 654 lipid molecules were identified, and 577 were RSD_30_number of QC samples of the SE group. So, the RSD_ Ratio (equal to RSD_30_number/total number) was 0.88 (more than 60%) in the QC samples of the SE group (Supplementary Figure S1C). This result demonstrated the high-quality data of the QC samples during the running process. Besides, Supplementary Figure S1D showed that the RSD_Ratio was 0.95 in the QC samples of the MY group. Therefore, the results suggested that the plasma lipidomic characteristics were reproducible and reliable in the two batches of MS experiments.

### Lipid identification and relative intensities of subclasses

According to the International Lipid Classification and Nomenclature Committee, lipid compounds are divided into eight categories, and each category contains several main classes. Each main class can be further divided into different subclasses according to differences in the polar heads. Each subclass can be divided into different molecular species according to differences in carbon chain saturation or length. Two batches of lipid species were directly identified using LipidSearch software from the accurate precursor *m/z* and MS/MS raw data with reference to a large-scale database. Also, statistical analysis was performed on several lipid molecules and subclasses after data pretreatment. For the batch of SE vs. H, a total of 494 unique lipid molecules were identified from the samples’ accurate masses and/or MS/MS data across 28 lipid subclasses via LipidSearch software (Supplementary Table S1). Because there are four levels of the identification results of lipids and the accuracy of A and B grades is relatively higher through the software, the number of lipid molecules of A/B/C levels is 227/81/180 in the MS data analysis, respectively. For the batch of MY vs. H, 491 unique lipids were identified by the software across 26 lipid subclasses (Supplementary Table S2), and the number of lipid molecules of A/B/C levels is 211/90/185.

The subclasses of the defined lipids could be classified into cholesteryl ester (ChE), zymosteryl (ZyE), SMs, Cer, sphingomyelin (phytosphingosine) (phSM), monoglycosylceramide (CerG1), diglycosylceramide (CerG2), sulfoquinovosyldiacylglycerol (SQDG), monogalactosyldiacylglycerol (MGDG), digalactosyldiacylglycerol (DGDG), triglycosyl-ceramide (CerG3), PCs, PEs, PIs, LPCs, dimethylphosphatidylethanolamine (dMePE), lyso-phosphatidylethanolamines (LPEs), PSs, phosphatidylinositol (PIP, PIP2), lysodimethylphosphatidylethanolamine (LdMePE), phosphatidylglycerols (PGs), phosphatidylethanol (PEt), cardiolipin (CL), triacylglycerols (TGs), diacylglycerols (DGs), fatty acid, (O-acyl)-1-hydroxy fatty acid (OAHFA), sphingoshine (So), coenzyme (Co), PA, and MG. Supplementary Figure S2 shows the statistical chart of lipid subclasses and the corresponding number of identified lipid molecules.

**Figure 1:**
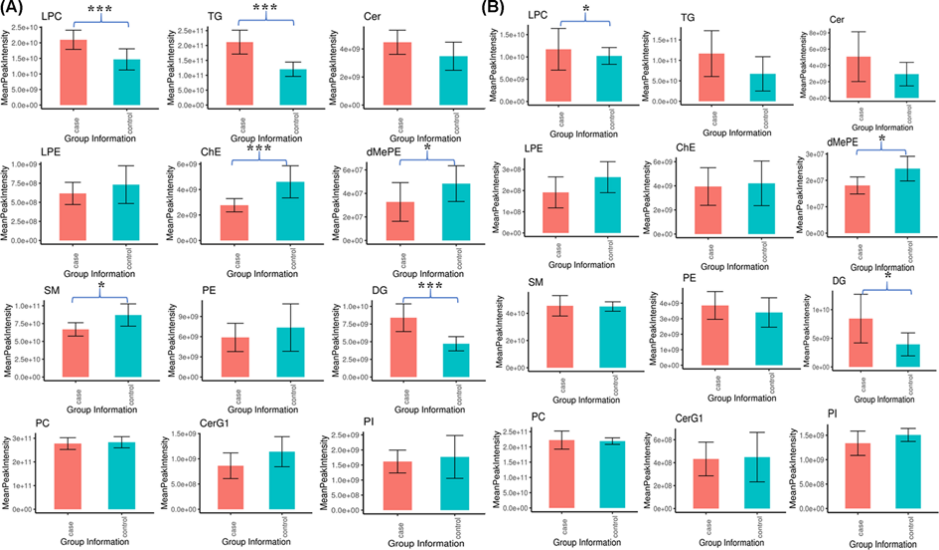
Relative Intensities of lipidomic subclasses in plasma between cured COVID-19 patients and healthy controls (**A**) SE vs. H, (**B**) MY vs. H. The data are expressed as the group mean value ± SD. * represents *P*<0.05, *** represents *P*<0.01.

Although there were some semi-quantitative differences in the lipids between different MS batches of the untargeted lipidomics, the batch effect did not affect the significances between groups. Because some subclasses in the MS identification process had only a few lipid species, 12 subclasses were selected for comparison of SE vs. H and MY vs. H, including LPC, TG, Cer, LPE, ChE, dMePE, SM, PE, DG, PC, CerG1, and PI. Figure 1 shows the relative amounts of these 12 lipid subclasses detected in each group at the lipid subclass level. Most of the significantly changed lipids were up-regulated in cured patients. A positive correlation existed between the alteration of lipids and deterioration of the disease. Lipid subclasses, including LPC, DG, and TG, were identified in higher abundance in SE vs. H and MY vs. H groups. The subclasses of ChE and dMePE were identified in lower abundance in SE vs. H groups.

### Differential lipid screening between cured COVID-19 patients and healthy controls

**Figure 2:**
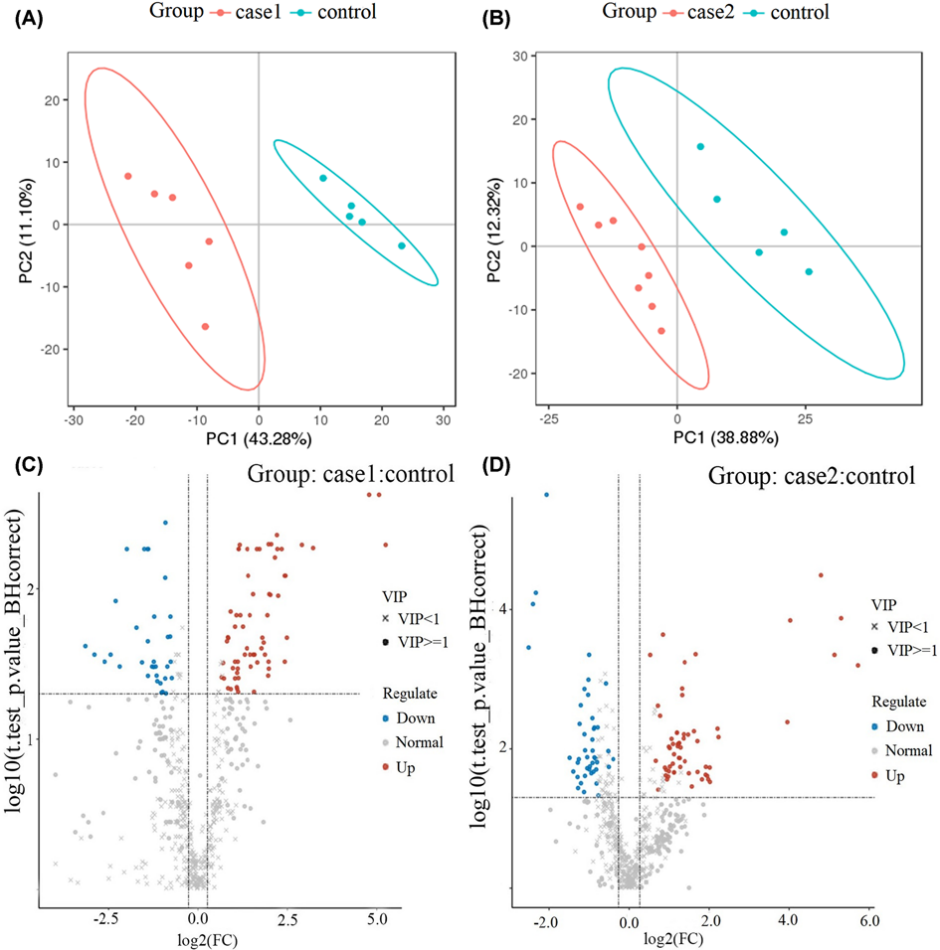
Multivariate and univariate analyses between cured COVID-19 patients and healthy controls (**A**) SE vs. H, (**B**) MY vs. H, of PLS-DA scores using Pareto scaling with mean centering. (**C**) SE vs. H, (**D**) MY vs. H, of Volcano Plot for visually displaying the differential lipid compounds. The blue are the down-regulated significantly differential lipid molecules, the red are the up-regulated significantly differential lipid molecules, the circle are the lipid molecules with VIP greater than or equal to 1, the ‘×’ are the lipid molecules with VIP less than 1, and the insignificant lipid molecules are marked as gray.

The plasma lipid differences among different groups were compared using score plots of the supervised PLS-DA to investigate the cluster data between cured COVID-19 patients and healthy control (Figure 2A for SE vs. H, Figure 2B for MY vs. H). PLS-DA models were established after the log2 logarithmic transformation and the Par scaling method. Seven-fold cross-validation was done when building the models, and 200 RPTs were performed on PLS-DA to assess the quality of the models. An apparent clustering trend was observed in different groups with acceptable R^2^Y (0.99 and 0.983) and Q^2^ (0.718 and 0.758), which indicated distinct plasma lipid profiles in response to COVID-19 virus infection and medical treatment, regardless of the SE or MY group. Meanwhile, the differential lipid molecules were visualized through the Volcano Plot (Figure 2C for SE vs. H, Figure 2D for MY vs. H), which indicates that univariate analysis was also suitable for the HRMS data.

**Figure 3:**
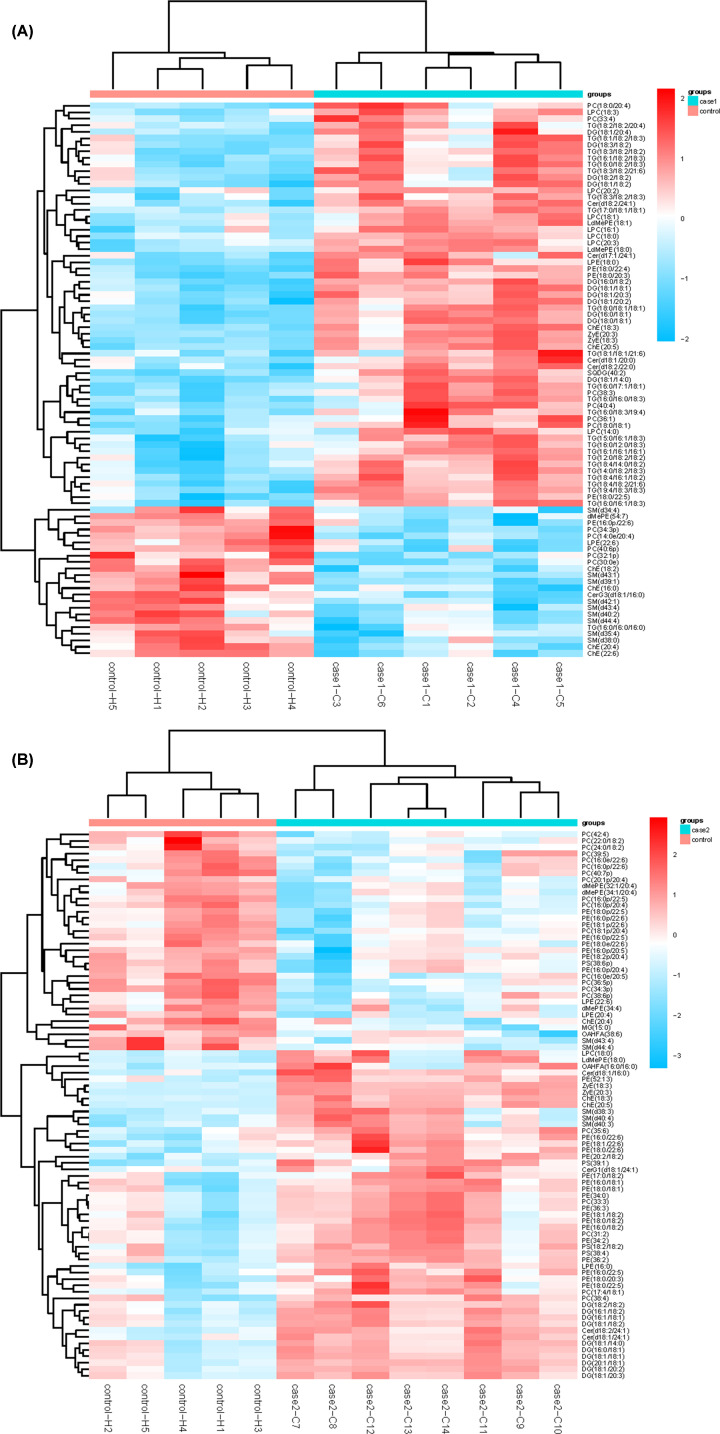
Heat map of differential lipid molecular species in cured COVID-19 patients compared with healthy controls (**A**) SE vs. H, (**B**) MY vs. H

**Figure 4:**
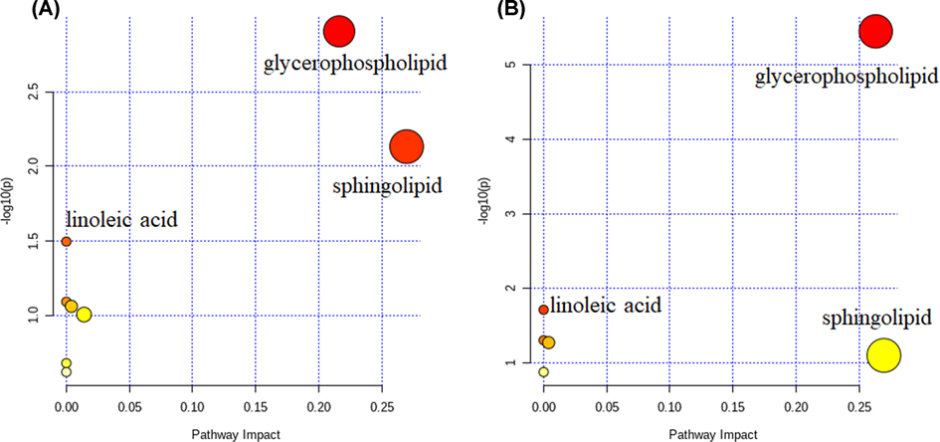
Lipidome KEGG pathway analysis of cured patients with COVID-19 (**A**) KEGG pathway analysis of differentiating lipids in the SE vs. H group. (**B**) KEGG pathway analysis of differentiating lipids in the MY vs. H group. The color bubbles represent the raw *P*-value, and the size of bubbles represents the number of counts.

Multivariate statistical analysis and univariate analyses were used together to screen the differential lipid molecules between groups. The alteration of plasma lipids was determined by VIP > 1.0 combined with a FC and Student’s *t* test, with FC ≥ 1.2 or ≤0.83 and *P*<0.05 set as the level of statistical significance (Table 2). According to the above criteria, there were 85 differential lipid molecules in SE vs. H groups listed in Supplementary Table S3, with 62 up-regulated (ZyE [20:3] of Max log2FC [5.25]) and 23 down-regulated (PC [34:3p] of Min log2FC [−3.16]). Supplementary Table S4 also lists 85 differential lipid molecules in MY vs. H groups with 51 up-regulated (ChE [18:3] of Max log2FC [5.72]) and 34 down-regulated (MG [15:0] of Min log2FC [−2.34]). Furthermore, the heat map of these differential lipid molecules is displayed in Figure 3. In the SE vs. H group, DG (18:3/18:2) exhibited the greatest log2FC (+2.43) in DGs, and TG (18:3/18:2/18:3) exhibited the greatest log2FC (+2.49) in all significantly altered TGs. In the MY vs. H group; however, DG (16:1/18:2) exhibited the greatest log2FC (+2.21) in all significantly altered DGs. Furthermore, differentiating lipids were subjected to KEGG pathway analysis via the Metaboanalyst online website. The results of the KEGG pathway analysis are presented in Table 3, which demonstrates that differential lipids were particularly enriched in GP metabolism and linoleic acid metabolism for both the SE and MY symptomatic groups (*P*<0.05). In the SE vs. H group, differentiating lipids were also significantly enriched in the SP metabolic signaling pathway (Figure 4).

**Table 2:** Overview of lipid molecules and total changed lipids

Batch	Lipid molecules	Differential lipids^*^	Up-regulated	Down-regulated	Max (Log2FC)	Min (Log2FC)
SE/H	494	85	62	23	5.25	−3.16
MY/H	491	85	51	34	5.72	−2.34

* *P*<0.05.

**Table 3:** KEGG pathway analysis of lipid metabolic pathways to SE/H and MY/H

Batch	Pathway	Total	Hits	Raw *P*	−log(p)	Holm adjust	FDR	Impact
SE/H	Glycerophospholipid metabolism	36	3	0.00123	2.9081	0.1038	0.103	0.2163
	Sphingolipid metabolism	21	2	0.00737	2.1324	0.6118	0.309	0.2697
	Linoleic acid metabolism	5	1	0.03188	1.4964	1	0.892	0
	α-Linolenic acid metabolism	13	1	0.08100	1.0915	1	1	0
	Glycosylphosphatidylinositol (GPI)-anchor biosynthesis	14	1	0.08698	1.0606	1	1	0.0039
	Glycerolipid metabolism	16	1	0.09883	1.0051	1	1	0.0140
	Arachidonic acid metabolism	36	1	0.20998	0.6778	1	1	0
	Steroid biosynthesis	42	1	0.24082	0.6183	1	1	0
MY/H	Glycerophospholipid metabolism	36	4	3.57E-06	5.4477	0.0003	0.0003	0.26332
	Linoleic acid metabolism	5	1	0.01923	1.716	1	0.807	0
	α-Linolenic acid metabolism	13	1	0.04935	1.3066	1	1	0
	Glycosylphosphatidylinositol (GPI)-anchor biosynthesis	14	1	0.05306	1.2752	1	1	0.0039
	Sphingolipid metabolism	21	1	0.07870	1.104	1	1	0.2697
	Arachidonic acid metabolism	36	1	0.13171	0.8803	1	1	0

## Discussion

The present study’s primary purpose was to generate a high-quality resource of lipidomic datasets between cured COVID-19 patients associated with different symptomatic or underlying diseases and healthy volunteers to understand the pathogenesis of COVID-19 better. The stable isotope addition strategy was also used in the untargeted lipidomics approach to analyze the plasma samples. The Q Exactive HRMS combines high sensitivity and accuracy and can accurately determine the composition of the precursor and product lipid ions. The LipidSearch software contains over 1700000 of lipid information and their predictive fragment ions. It can provide powerful bioinformatics capabilities for the automated identification of lipids and integration of an entire dataset into a concise report showing statistical differences between sample groups. MS2 results in the LC-HRMS analysis matched to lipid datasets for lipid identification, which was conducted by LipidSearch software against databases to improve the confidence of lipidomic identification. After lipidomic feature extraction and alignment, the full-scan MS results in the LC-HRMS analysis were used in the multivariate/univariate statistical analysis to extract statistically significant lipidomic features between groups. The endogenous lipids as the potential biomarkers for disease prognosis are mapped on to metabolic pathways to reveal possible pathway dysregulation during viral infection and medical treatment.

Our lipidomics results showed that over 50 lipids, including TGs, DGs, and LPCs, were up-regulated in the plasma of both the SE and MY groups of cured COVID-19 patients. This result is probably because of damage to the liver, which is consistent with the observations that many COVID-19 patients showed liver function abnormalities [9,20]. Concerning the alteration of plasma TGs in the SE vs. H groups, an interesting finding was that most polyunsaturated fatty acid-containing TGs were increased significantly, such as TG (18:3/18:2/18:3) and TG (18:3/18:2/18:2) (Figure 3A). This increased proportion of polyunsaturated fatty acid in TGs could be attributed to an increase in the relative amount of polyunsaturated fatty acids available to the liver for TGs’ assembly rather than the release of saturated or monounsaturated fatty acids from adipose tissue, such as TG (16:0/16:0/16:0) [28,29]. Furthermore, the lipids involved in the glycerol metabolic pathway, which maintains the balance of the energy metabolites in the body, suggests that SARS-CoV-2, like many other viruses, probably hijacks the cellular metabolism [30].

SM, LPE, dMePE, and ChE were down-regulated in cured COVID-19 patients compared with healthy controls. Reports suggest that SPs and glycosphingolipids might play essential roles in the early development of enveloped viruses [31]. PEs may contribute to driving the suppression of inflammatory responses in human cells by a medically and globally important dengue fever virus [32]. Low total ChE can usually be caused by liver damage, such as viral hepatitis, liver cirrhosis, and liver cancer [33]. Therefore, the dramatic reduction of such lipids was also consistent with the hepatic impairment associated with COVID-19, especially for patients in the SE group. The differential lipids were particularly enriched in GPs, SP, and linoleic acid metabolism for pathway analysis. GPs and SPs are essential components of biomembranes, which mediate signal transduction and immune activation processes. Furthermore, SPs regulate diverse processes, including growth regulation, cell migration, adhesion, apoptosis, senescence, and inflammatory responses [34].

The lipidomic alternations in patient plasma mainly reflect the systematic responses of SARS-CoV-2 to the metabolism of diverse cell types and organs. So, the interpretations of our lipidomic datasets should also be combined with other types of systematic studies, such as transcriptomic and proteomic studies, clinical observations, and laboratory examinations, to provide more comprehensive disease information. Such integration not only would help us to understand the effects of SARS-CoV-2 on specific cells and tissues, but also might explain why some patients are vulnerable to COVID-19 while others are not. However, a major limitation of the study was that age was not taken into account when comparing SE group with healthy controls (less than 35 years old), since age may also be an important factor relating to lipid changes. Therefore, such limitation of lipidomics data should be paid more attention to SE group, especially for differential analysis. Another limitation of the present study was that the sample size was rather small, which only comprised five to eight participants in each group. So the individual factors such as underlying diseases may affect the results of statistical analysis. Like the observation of lipidomic alterations, it is apparent that these cured patients, whether they continue to experience severe or mild symptoms, were not fully recovered from the aftermath of COVID-19 from the aspects of lipid metabolism. Therefore, these discharged patients should continue with consolidation therapy to recover fully from the physiological effects of COVID-19.

## Conclusion

In the present study, untargeted lipidomics for high coverage of lipid characterization was implemented using UPLC with the Q Exactive HRMS and bioinformatics tools. The statistical and differential analyses of plasma lipids were also performed between healthy controls and cured COVID-19 patients, who were further divided into the SE and MY groups. Our results indicated that lipidomic alteration was dramatically changed, even in the patients who attained the discharged standards. So, it is better to continue the subsequent physical therapy and consolidation treatment. Probably because of relatively small sample size and no fully consideration of age factor, more rigorous study design may be carried out in bigger cohorts in the future. Such an MS-based lipidomics strategy can help obtain highly valuable resources to improve understanding of host metabolic responses associated with COVID-19. This information will broaden our knowledge about the pathogenesis of COVID-19 and accelerate the identification of disease biomarkers.

## Supplementary Material

Supplementary Figures S1-S2

Supplementary Tables S1-S4

## Data Availability

The mass spectrometry data have been deposited in iProX (www.iprox.org) with ID IPX0002664000 and the ProteomeXchange Consortium (http://proteomecentral.proteomexchange.org) via the iProX partner repository [35] with the dataset identifier PXD023154.

## Abbreviations

ACN: acetonitrile
Cer: ceramide
CerG1: monoglycosylceramide
ChE: cholesteryl ester
CL: cardiolipin
Co: coenzyme
COVID-19: novel coronavirus disease 2019
DG: diacylglycerol
dMePE: dimethylphosphatidylethanolamine
EVD: Ebola virus disease
FA: fatty acyl
FC: fold-change
GL: glycerolipid
GP: glycerophospholipid
H: healthy control
HRMS: high-resolution mass spectrometry
IPA: isopropanol
KEGG: Kyoto Encyclopedia of Genes and Genomes
LPC: lyso-phosphatidylcholine
LPE: lyso-phosphatidylethanolamine
max IT: maximum ion injection time
MG: monoglyceride
MTBE: methyl-tert-butyl ether
MY: young people with mild symptoms
PA: phosphatidic acid
PC: phosphatidylcholine
PCA: principal component analysis
PE: phosphatidylethanolamine
PEt: phosphatidylethanol
PG: phosphatidylglycerol
PI: phosphatidylinositol
PLS-DA: partial squared discriminant analysis
PS: phosphatidylserine
QC: quality control
RPT: response permutation testing
SARS-CoV-2: severe acute respiratory syndrome coronavirus 2
SE: severe symptom or elderly patient
SM: sphingomyelin
So: sphingoshine
SP: sphingolipid
TG: triacylglycerol
VIP: variable importance of projection
ZyE: zymosteryl
