# Supplementary material for: Lipidomic alteration of plasma in cured COVID-19 patients using ultra high-performance liquid chromatography with high-resolution mass spectrometry
Source: Biosci Rep. 2021 Mar 5;41(3):BSR20204305. doi: 10.1042/BSR20204305 (PMC7937909; doi:10.1042/BSR20204305)
Supplement: Supplementary Figures S1-S2 [file BSR-2020-4305_supp.pdf]

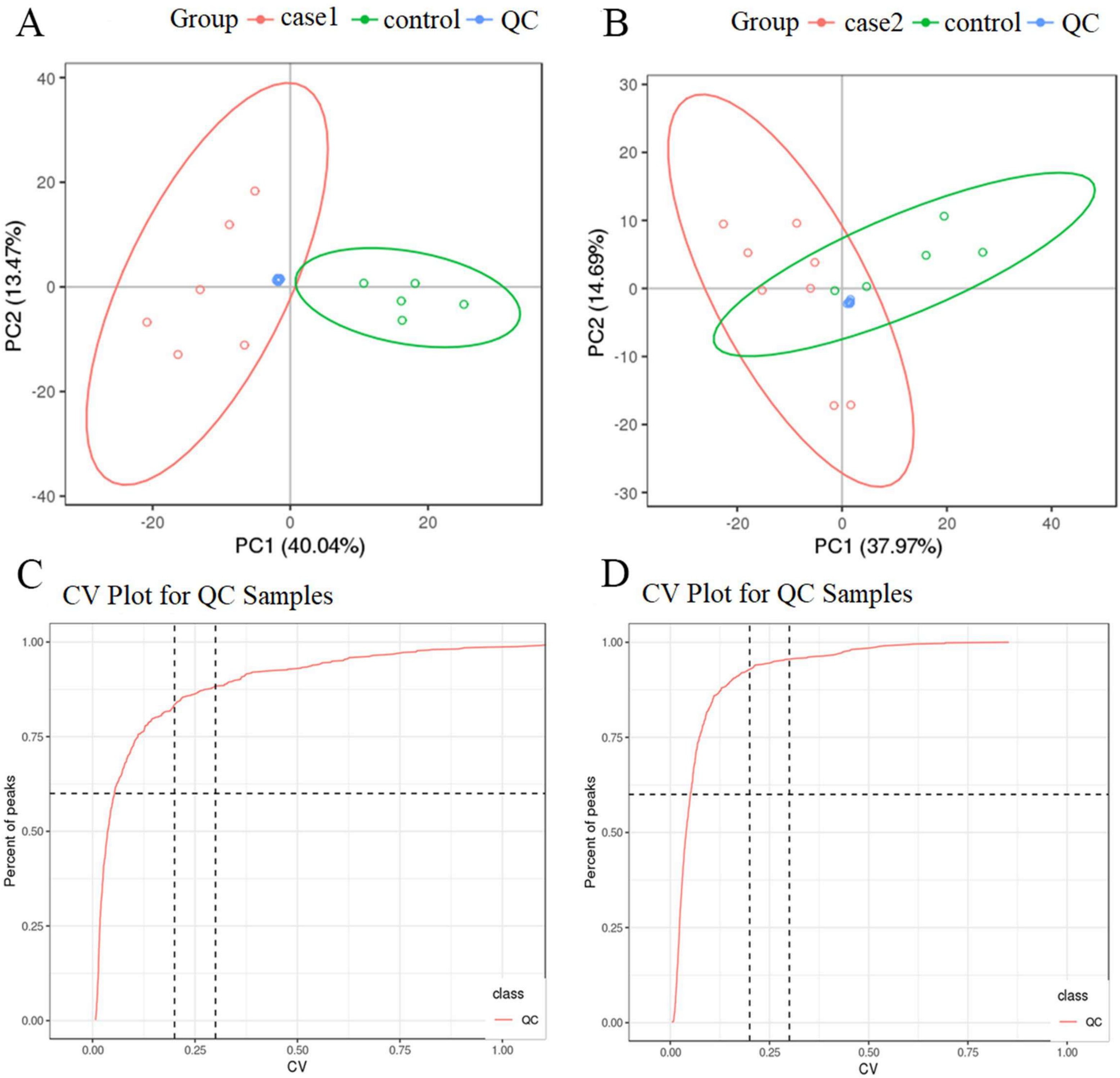
A
Group case1 control QC
PC1 (40.04%)
PC2 (13.47%)
B
Group case2 control QC
PC1 (37.97%)
PC2 (14.69%)
C
CV Plot for QC Samples
Percent of peaks
CV
class
QC
D
CV Plot for QC Samples
Percent of peaks
CV
class
QC

Figure S1. PCA score plot of cured COVID-19 patients (A: SE group, B: MY group), healthy controls, and pooled QCs. The CV distribution of lipid molecules in QC samples of the SE group (C) and the MY group (D).

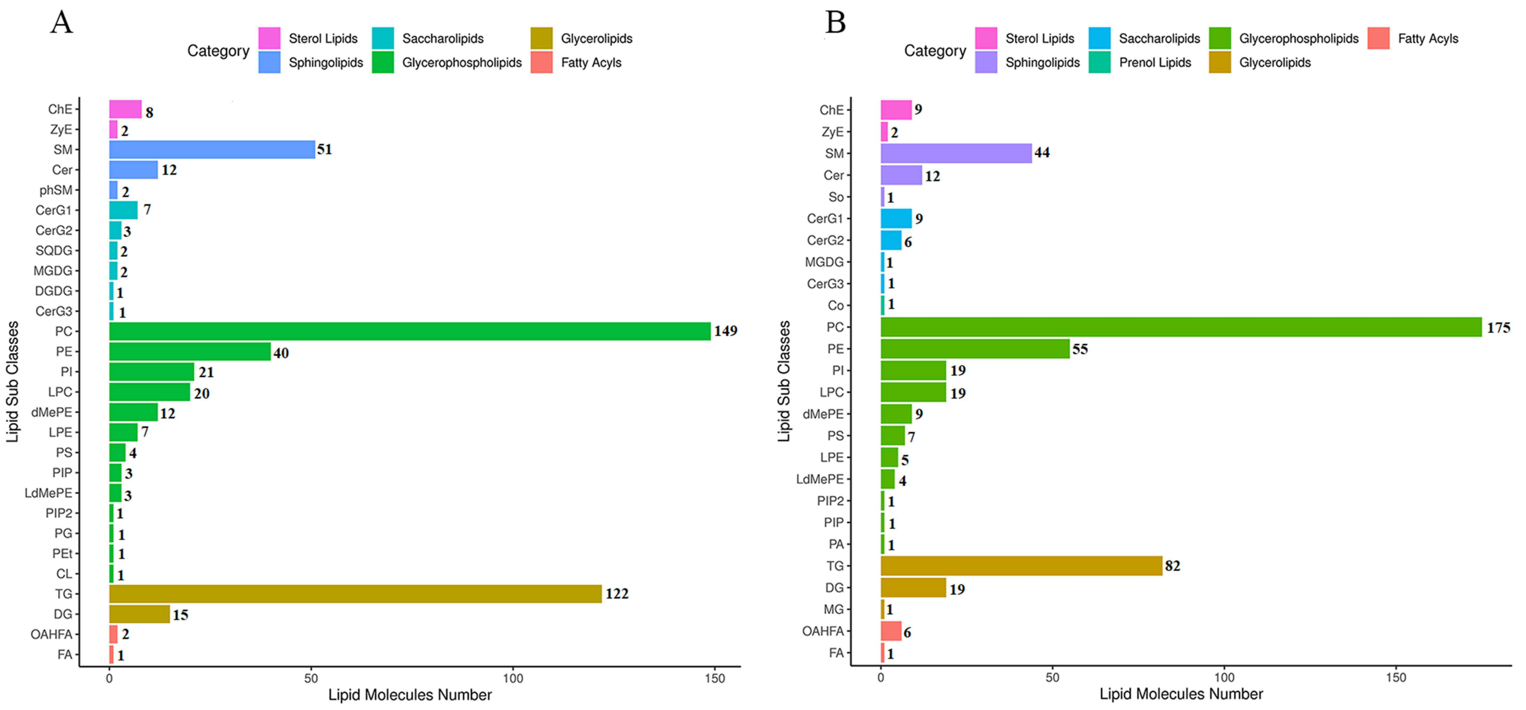
A
Category
Sterol Lipids
Sphingolipids
Saccharolipids
Glycerophospholipids
Glycerolipids
Fatty Acyls
ChE 8
ZyE 2
SM 51
Cer 12
phSM 2
CerG1 7
CerG2 3
SQDG 2
MGDG 2
DGDG 1
CerG3 1
PC 149
PE 40
PI 21
LPC 20
dMePE 12
LPE 7
PS 4
PIP 3
LdMePE 3
PIP2 1
PG 1
PEt 1
CL 1
TG 122
DG 15
OAHFA 2
FA 1
Lipid Sub Classes
0
50
100
150
Lipid Molecules Number
B
Category
Sterol Lipids
Sphingolipids
Saccharolipids
Prenol Lipids
Glycerophospholipids
Glycerolipids
Fatty Acyls
ChE 9
ZyE 2
SM 44
Cer 12
So 1
CerG1 9
CerG2 6
MGDG 1
CerG3 1
Co 1
PC 175
PE 55
PI 19
LPC 19
dMePE 9
PS 7
LPE 5
LdMePE 4
PIP2 1
PIP 1
PA 1
TG 82
DG 19
MG 1
OAHFA 6
FA 1
Lipid Sub Classes
0
50
100
150
Lipid Molecules Number

Figure S2. Statistical chart of lipid subclasses and corresponding lipid molecules.(A) SE group, (B) MY group. The vertical axis, horizontal axis, and different colors correspond to the lipid subclasses, the number of lipid molecules identified in each subclass, and different categories.
